# Number of vaginal lactobacilli in postmenopausal women with vaginal atrophy before and after treatment with erbium–YAG laser: a randomized sham-controlled trial

**DOI:** 10.1186/s12905-023-02590-y

**Published:** 2023-09-26

**Authors:** Nuttanun Panyawongudom, Krasean Panyakhamlerd, Ammarin Suwan

**Affiliations:** Department of Obstetrics and Gynecology, Faculty of Medicine, Chulalongkorn University, Bangkok, Thailand

**Keywords:** Erbium**–**YAG laser, Vaginal lactobacilli, Randomized sham-controlled trial, Menopause, Vaginal atrophy, Vaginal pH

## Abstract

**Primary objective:**

To evaluate the effect of erbium**–**YAG laser on the number of vaginal lactobacilli in postmenopausal women.

**Secondary objectives:**

To evaluate the effect of erbium**–**YAG laser on vaginal atrophy symptoms and vaginal pH in postmenopausal women.

**Materials and methods:**

A total of 44 postmenopausal women who met the inclusion criteria were randomized in the laser group (n = 22) and sham group (n = 22). Vaginal lactobacilli grading, vaginal pH, vaginal atrophy score, and vaginal atrophy symptoms were assessed before and after treatment with erbium**–**YAG laser for two consecutive times, with a four-week interval; the results were compared with the effects of the sham procedure. Any adverse events after the treatment were recorded.

**Results:**

A total of 44 women were included, and five were lost to follow-up. Compared with sham procedure, vaginal lactobacilli grading improved in the laser group (5/20 in the laser group and 1/19 in the sham group). However, the improvement did not reach statistical significance (adjusted odds ratio = 5.32, 95% CI = 0.5–56.21). Vaginal atrophy symptoms measured by the visual analog scale (VAS) and vaginal pH were improved in both groups without a statistically significant difference between the two groups. Vaginal “dryness” VAS and vaginal atrophy score after treatment were significantly lowered in the laser group compared with the sham group.

**Conclusions:**

This study showed an improvement in vaginal lactobacilli grading after vaginal laser treatment. However, the difference in vaginal lactobacilli grading after treatment in both groups was not statistically significant.

## Introduction

Vaginal atrophy or genitourinary syndrome of menopause (GSM) is commonly found in postmenopausal women. About 50% of postmenopausal women suffer from vaginal atrophy symptoms [1]. This condition is due to low estrogen levels after menopause [2]. The diagnosis of vaginal atrophy is clinical, which is manifested by vaginal dryness, irritation, pain, dyspareunia, and urinary symptoms [3].

Vaginal atrophy can be treated by either local hormonal treatment or non-hormonal treatment. At present, vaginal estrogen therapy is a standard treatment for vaginal atrophy. However, caution must be observed in hormone-sensitive cancer survivors [4]. Moreover, a number of patients denied hormonal treatment. Thus, non-hormonal treatments, including vaginal erbium**–**YAG laser, have been considered.

Vaginal laser is a novel treatment for vaginal atrophy. Two types of laser can be used for intravaginal therapy, namely, CO_2_ and erbium**–**YAG laser [5]. Erbium–YAG laser has 15 times more water absorption than CO_2_ laser, thereby causing less penetration, faster tissue healing phase, fewer side effects, and lesser pain compared to the CO_2_ laser [4].

Vaginal laser has a thermal effect on vaginal epithelium, resulting in the expression of heat-shock proteins, which stimulate growth factor activities, neovascularization, neocollagenesis, and extracellular matrix formation as well as increased vaginal thickness and elasticity. However, the thermal effect of erbium–YAG laser is less than that of CO_2_ laser, thereby causing no tissue damage [4].

Many prospective studies reported that vaginal erbium–YAG laser can improve vaginal atrophy symptoms, and its effect is comparable to that of vaginal estrogen therapy [6–8].  A systematic review and meta-analysis of laser therapy for GSM published in 2017, included 14 studies of CO_2_ and erbium–YAG laser, showed that vaginal laser improved GSM symptoms, female sexual function index, vaginal health index score, and vaginal maturation value. However, the quality of evidence was “low” or “very low” because no randomized sham-controlled study can decrease the bias and placebo effect [5].

Vaginal lactobacilli are normal vaginal flora that can produce antimicrobial compounds, such as hydrogen peroxide and lactic acid, and compete with pathogens for vaginal adherence [9].  A number of vaginal lactobacilli increase after vaginal epithelial maturation because of an increase of glycogen storage in superficial vaginal epithelium [10, 11]. Therefore, it can be used as an objective measurement of vaginal atrophy. As vaginal lactobacilli decrease during postmenopausal period, the postmenopausal women are at increased risk of vaginal pathogenic bacteria and mycotic infection.

A prospective study of vaginal CO_2_ laser and vaginal flora reported a significant increase of vaginal lactobacilli and a significant decrease of vaginal pH after three applications of CO_2_ laser [9] . owever, no study has been conducted on the effect of vaginal erbium–YAG laser and number of vaginal lactobacilli to date.

This study aimed to measure the effect of vaginal erbium–YAG laser on a number of vaginal lactobacilli. We also evaluate vaginal pH, vaginal atrophy symptoms, and adverse effects after vaginal erbium–YAG laser treatment.

## Materials and methods

This randomized sham-controlled study was conducted at King Chulalongkorn Memorial Hospital, Bangkok, Thailand, between May 2019 and September 2020. The target population was postmenopausal women attending gynecology clinic or gender health clinic with vaginal atrophy symptoms (dryness, dyspareunia, irritation, and pain). The inclusion criteria were as follows: postmenopausal women with at least one symptom of moderate-to-severe vaginal atrophy and age more than 40 years. (Moderate-to-severe vaginal atrophy was defined by the most bothersome symptom score at least “2” in at least one vaginal atrophy symptom [0 = no symptom, 1 = mild, 2 = moderate, 3 = severe]). As the inclusion criteria of our study were women older than 40 years, the postmenopausal women in this study included natural menopause, surgical menopause, primary ovarian insufficiency, and participants with history of chemoradiation therapy for cancers. Both sexually active and sexually inactive women were included in this study. The exclusion criteria were as follows: history of hormonal therapy, vaginal lubricants, vaginal moisturizers, or spermicide in previous three months; active or recent genitourinary tract infection in previous one month; abnormal vaginal bleeding; genital organ prolapse stage II; history of vaginal surgery in previous three months; and previous vaginal laser treatment.

This study was conducted at King Chulalongkorn Memorial Hospital (KCMH), Bangkok, Thailand, between May 2019 and September 2020. The study was not advertised. The primary outcome of this study was to evaluate the effect of erbium YAG laser on the number of vaginal lactobacilli. The secondary outcomes were to evaluate the effect of erbium YAG laser on vaginal atrophy symptoms and vaginal pH.

After obtaining written informed consent, all participants were interviewed to collect demographic data and randomized into the laser group and sham group using block-of-four randomization with a sealed envelope. Participants in the laser group were treated with erbium in yttrium aluminum–garnet crystal (Er:YAG) laser (Fotona Smooth™ XS, Fotona, Ljubljana, Slovenia) at a wavelength of 2940 nm, with a spot size of 7 mm, frequency of 1.6 Hz, and laser energy of 6.0 J/cm [2], two times every four weeks. Participants in the sham group were treated with two sham operations every four weeks. Sham operation was conducted while the patient was in the lithotomy position, and the same equipment as vaginal erbium laser was used, but no laser energy was emitted from the equipment. The duration of sham operation was equal to that of vaginal erbium laser treatment or about 5 min. All participants were appointed for follow-up visit four weeks after the second laser or sham application. Vaginal atrophy symptoms were measured by using the visual analog scale (VAS) in every visit. Vaginal swabs were collected from the posterior fornix, and vaginal pH was measured using a pH indicator strip applied to the lateral vaginal wall. Vaginal atrophy score (Table 1) was assessed in every visit. Vaginal swabs were sent to a microbiologist to evaluate the vaginal lactobacilli grading. Vaginal lactobacilli were reported into four grades in accordance with the number of lactobacilli per high power field (grade 1: < 6/HPF, grade 2: 6–20/HPF, grade 3: 21–50/HPF, and grade 4: > 50/HPF). All participants were not received reimbursement to be enrolled but the authors provided travelling expenses for each follow-up visit.

**Table 1:** Assessment of vaginal atrophy score

	Not present (0)	Mild (1)	Moderate (2)	Severe (3)
Dryness	Normal lubrication	Slightly decreased	Minimal lubrication	Dry
Rugae	Normal number and depth	Reduced rugae	Rare rugae	Smooth vagina
Pallor	Normal pink	Light pink	Very pale	White/deep red
Petechiae	None	Bleeds on scraping	Bleeds on contact	Clearly seen
Mucosal elasticity	Normal	decreased	None	Stenosis

For sample size calculation, two independent proportion formulas were used. The sample size was calculated using data obtained from our pilot study. The proportion in the study group and control group was 0.4 and 0.9, respectively. (The result of sample size calculation was the proportion of participants with grade 1 vaginal lactobacilli after vaginal erbium laser treatment and after sham operation). Using 5% type I error and 20% type II error, the calculated sample size was 17 per group. Adding 20% drop out, a total of 42 participants were needed.

The randomization sequence was generated by computer generated block of 4 randomization and the allocation concealment was done by sequentially numbered sealed envelopes. The first author (N.P.) kept the randomization schedule. All participants were blinded during the study period. All vaginal swab slides were sent to a microbiologist with identification numbers that did not mention about the study group. Therefore, a microbiologist who evaluated vaginal lactobacilli grading was also blinded. However, all the vaginal laser and sham applications were performed by the first author (N.P.) and those procedures cannot be blinded to the provider.

This study was funded by Ratchadapisek Sompot and approved by the Institutional Review Board of Faculty of Medicine, Chulalongkorn University. This study was registered in Thai Clinical Trial Registry on 30/10/2019 (TCTR20191030001; https://www.thaiclinicaltrials.org/export/pdf/TCTR20191030001).

Statistical analysis was performed by SPSS version 22.0. For descriptive statistics, the number, percentage, and mean ± standard deviation (SD) were used for continuous data, while the number and percentage were used for categorical data. For analytical statistics, repeated measures ANOVA and Friedman test were used for continuous and categorial data, respectively. A *p* value of < 0.05 was considered statistically significant.

## Results

**Fig. 1:**
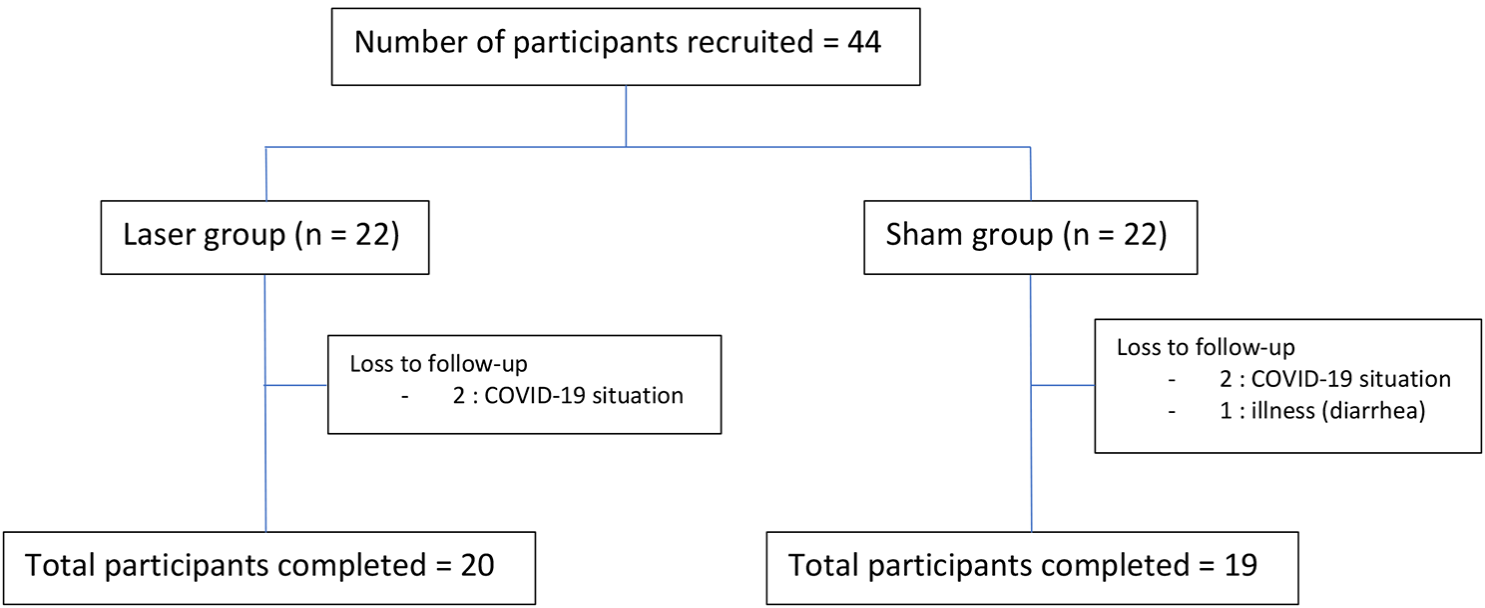
Consort diagram of the recruitment and drop out of the participants in the laser group and sham group

A total of 44 participants were recruited and randomized to the laser group and sham group, with 22 participants in each group. Of the participants, five were lost to follow-up, and a total of 39 participants completed the study (the detail was shown by a consort diagram in Fig. 1). Table 2 shows similar baseline characteristics of participants in the laser group and sham group, but the participants in the laser group were younger than those in the sham group (the mean age in the sham group was 60.4, and the mean age in the laser group was 55.5). No significant difference in parity, menopausal age, year since menopause, baseline vaginal atrophy symptoms measured by VAS, baseline vaginal pH, and baseline vaginal atrophy score was observed among participants in both groups. In addition, no significant difference in BMI was found between the two groups. Similar baseline vaginal lactobacilli were observed between the laser group and sham group. The most prominent symptom of the participants was dyspareunia (54.5% in both groups).

**Table 2:** Baseline characteristics of the subjects in the laser group and sham group

	Laser group (mean ± SD)	Sham group (mean ± SD)	*p* value
Age (year)	55.5 ± 7.6	60.4 ± 6.6	0.03
Parity	1.5 ± 1.0	1.8 ± 1.1	0.47
Menopausal age (year)	47.3 ± 4.3	49.7 ± 5.3	0.10
Year since menopause (year)	9.0 ± 6.4	10.7 ± 7.1	0.41
Vaginal atrophy symptoms (VAS)	17.5 ± 8.6	16.3 ± 7.5	0.62
Vaginal pH	6.3 ± 1.4	6.6 ± 1.2	0.35
Vaginal atrophy score	6.5 ± 2.9	7.9 ± 2.9	0.13
	Laser group N (%)	Sham group N (%)	*p* value
BMI			1.00
< 18.5	1(4.5)	0(0)	
18.5–24.99	12(54.6)	13(59.1)	
25–29.99	7(31.8)	7(31.8)	
≥ 30	2(9.1)	2(9.1)	
Lactobacilli grade			0.55
1	17(77.3)	21(91)	
2	2(9.1)	1(4.5)	
3	1(4.5)	0(0)	
4	2(9.1)	1(4.5)	
Most symptoms			0.27
Dryness	8(36.5)	4(18.3)	
Dyspareunia	12(54.5)	12(54.5)	
Irritation	1(4.5)	5(22.7)	
Pain	1(4.5)	1(4.5)	

Table 3 shows vaginal lactobacilli grading in each visit of both groups. Visit 1 is pre-treatment. Visits 2 and 3 are after the first and second treatments, respectively. In the laser group, the grade of vaginal lactobacilli was significantly higher after the second treatment (*p* = 0.02). However, no statistically significant change in vaginal lactobacilli was observed after two sham procedures. In comparison between the two groups, using logistic regression for calculation of odds ratio (OR) of improvement after two laser treatments, the adjusted OR for age was 5.32 (95% CI = 0.5–56.21, *p* = 0.17). Therefore, compared with the sham group, the improvement of vaginal lactobacilli grading of participants in the laser group did not reach statistical significance.

**Table 3:** Vaginal lactobacilli grading, vaginal atrophy symptoms (VAS), vaginal atrophy score, and vaginal pH in each visit of participants in the laser group and sham group

	Laser group				Sham group			
**Lactobacilli grade**	Grade 1	Grade 2	Grade 3	Grade 4	Grade 1	Grade 2	Grade 3	Grade 4
Visit 1	15	2	1	2	17	1	0	1
Visit 2	16	0	1	3	16	2	0	1
Visit 3	13	1	1	5	17	1	1	0
p value	0.02				0.72			
**Vaginal atrophy symptoms (VAS)**	**Laser group (mean ± SD)**				**Sham group (mean ± SD)**			
Visit 1	17.5 ± 8.6				16.3 ± 7.5			
Visit 2	10.5 ± 8.7				13.8 ± 8.9			
Visit 3	6.8 ± 5.5				10.0 ± 8.2			
p value	< 0.001				0.08			
**Vaginal atrophy score**								
Visit 1	6.5 ± 2.9				7.9 ± 2.9			
Visit 2	4.3 ± 2.1				6.6 ± 2.3			
Visit 3	3.9 ± 2.4				5.6 ± 1.9			
p value	< 0.001				< 0.001			
**Vaginal pH**								
Visit 1	6.3 ± 1.4				6.6 ± 1.2			
Visit 2	6.0 ± 1.3				6.6 ± 1.2			
Visit 3	6.1 ± 1.2				6.2 ± 1.1			
p value	0.20				0.15			

Vaginal atrophy symptoms, which were measured by VAS, were significantly decreased after treatment in the laser group. However, no statistical difference in vaginal atrophy symptoms was observed after sham operations. Table 3 also demonstrates that the vaginal atrophy score after treatment was significantly lowered in the laser group and sham group (*p* < 0.001). In addition, no statistically significant difference in vaginal pH before and after treatment was observed in both groups (*p* = 0.20 and 0.15 in the laser group and sham group, respectively).

In determining secondary outcomes, mixed-effect multiple linear regression was used, and it showed a significant change in vaginal dryness (mean change = − 2.28, *p* = 0.01) and vaginal atrophy score (mean change = − 1.49, *p* = 0.03) before and after treatment in the laser group as compared with the sham group. The changes in vaginal pH, total vaginal atrophy symptoms, pain, irritation, and dyspareunia were not statistically significant. The secondary outcome results are shown in Table 4. For the adverse event, only one participant in the sham group complained about pain during operation, but it was improved after the operation.

**Table 4:** Secondary outcome adjusted with time and age because of imperfect randomization

Outcome	Mean change	*p* value	95% CI
VAS	−2.73	0.15	−6.44 to 0.98
Dryness	−2.28	0.01	−4.11 to − 0.46
Irritation	−0.36	0.57	−1.64 to 0.91
Soreness	−1.18	0.07	−2.44 to 0.08
Dyspareunia	−1.50	0.09	−3.22 to 0.22
Vaginal atrophy score	−1.49	0.03	−2.82 to − 0.16
pH	−0.0	0.86	−0.61 to 0.51

## Discussion

Vaginal erbium–YAG laser is a novel treatment for vaginal atrophy, which is currently known as GSM. Literature showed the effectiveness and safety of intravaginal laser therapy, while erbium–YAG laser is considered as a safer option as compared with fractional CO_2_ laser because of its non-ablative effect [4–8].

The mechanism of action of erbium–YAG laser is its photothermal effect on the vaginal epithelium, which causes superficial tissue shrinkage, neovascularization, and neocollagenesis, resulting in vaginal tissue thickening, and increases vaginal elasticity [4].

Based on the literature, few studies have reported an improvement of vaginal atrophy symptoms after vaginal erbium–YAG laser treatment [6–8]. However, no study has been conducted on vaginal erbium–YAG laser and vaginal lactobacilli, which can be used as an objective measurement. Vaginal lactobacilli are vaginal normal flora that dynamically changed with vaginal maturation, and they play a role in maintaining vaginal acidity and compete with pathogens [9, 10, 12].

To our knowledge, this study is the first randomized sham-controlled study to assess the effect of vaginal erbium–YAG laser on vaginal lactobacilli, vaginal pH, and vaginal atrophy symptoms. This study reports the objective and subjective measurements of vaginal atrophy after vaginal erbium–YAG laser treatment. The results show an increase of vaginal lactobacilli grading after two laser applications. The increase of vaginal lactobacilli grading can be described by the effect of vaginal laser on vaginal epithelial maturation, that is, superficial cells of the vaginal epithelium are increased after treatment with vaginal laser. The superficial cells are glycogen-rich cells, and lactobacilli use glycogen as their nutrients and produce lactic acid. Moreover, vaginal atrophy symptoms assessed by VAS and vaginal atrophy scores were improved after laser treatments. The reduction in VAS score in this study is similar to the results of previous studies [5–8, 13–15]. However, compared with several reports,[9, 16] no statistically significant difference in vaginal pH before and after vaginal laser applications is observed in this study. This result can be due to the majority of participants with grade 1 vaginal lactobacilli after vaginal laser treatment. Their vaginal epithelium contains only few lactobacilli that cannot produce enough lactic acid to lower vaginal pH.

A recent large prospective observational study of fractional CO_2_ laser conducted in Italy has shown a significant improvement of vaginal lactobacillus species at four weeks after CO_2_ laser treatment [17]. This study also demonstrates the improvement of vaginal atrophy symptoms indicated by the most bothersome symptoms. However, compared with the result of our study, a significant improvement in vaginal pH was observed after the treatment. This result can be explained by the differences in laser energies and percentage of participants with lactobacillus-predominated vaginal wet smear.

The safety of intravaginal laser therapy is a major concern. The result from previous studies showed only few minor adverse events after vaginal laser application [5, 8, 16, 18] In our study, only one patient in the sham group complained about pain after treatment, which was improved during the follow-up period. In addition, no side effects were reported after treatment in the laser group. Therefore, the safety result from our study is concordant with previous studies,[5, 8] confirming the short-term safety of vaginal erbium–YAG laser treatment. However, long-term follow-up is necessary to evaluate the long-term safety of this treatment.

This study has several strengths. First, this study has a randomized sham-controlled design, which can diminish the bias and placebo effect. Second, our study evaluates the subjective (VAS score) and objective (vaginal lactobacilli, vaginal pH, and vaginal atrophy score) outcomes of vaginal atrophy after vaginal laser treatment.

However, this study also has a limitation, which is the different baseline characteristics between the laser group and sham group (participants in the laser group were younger that those in the sham group), which results from imperfect randomization. Moreover, the age difference between groups can have an impact on vaginal lactobacilli grading. Therefore, the main findings of this study were the improvement of vaginal atrophy symptoms and vaginal atrophy score after the vaginal laser treatments.

Another limitation of our study was lacking an active control group (such as vaginal estrogen, vaginal lactobacilli, or other non-hormonal treatments). Thus, for clinical implication, clinicians should be acknowledged this limitation. However, as this study showed the improvements of vaginal atrophy after the vaginal erbium laser procedure, it might lead to further clinical trials with an active control group.

A significant number of participants were lost to follow-up during the study period because of the COVID-19 situation and personal illness that is not related to the procedure. However, the number of participants who were lost to follow-up was similar in the laser and sham groups (2/22 and 3/22, respectively). Therefore, the effect of loss to follow-up might not interfere with the outcome of this study.

For further research, increasing the sample size or vaginal laser applications might show a statistically significant improvement of subjective and objective measurements of vaginal atrophy after vaginal erbium laser treatment. Moreover, further research may focus on the possible benefits of the vaginal erbium laser in other study populations such as menopause women after ovarian tissue cryopreservation and transplantation or patients with refractory lower urinary tract dysfunction [19, 20].

## Conclusion

No statistically significant difference in vaginal lactobacilli grading was observed before and after treatment with erbium–YAG laser as compared with the sham procedure. However, the grade of lactobacilli tended to increase after two applications of vaginal laser. Vaginal “dryness” was improved significantly after treatment with erbium–YAG laser. Furthermore, no serious adverse events were reported.

## Data Availability

All data and materials are described in the manuscript and tables.
